# AKR1B10 inhibits the proliferation and migration of gastric cancer via regulating epithelial-mesenchymal transition

**DOI:** 10.18632/aging.203538

**Published:** 2021-09-22

**Authors:** Xinyu Shao, Jue Wu, Shunying Yu, Yuqing Zhou, Chunli Zhou

**Affiliations:** 1 Department of Gastroenterology, The Affiliated Suzhou Hospital of Nanjing Medical University, Suzhou Municipal Hospital, Gusu School, Nanjing Medical University, Suzhou, Jiangsu, China; 2 Department of Obstetrics and Gynecology, The Suzhou Dushu Lake Hospital, Suzhou, Jiangsu, China

**Keywords:** AKR1B10, gastric cancer, proliferation, migration, EMT

## Abstract

Gastric cancer (GC) is a common malignancy around the world with a poor prognosis. Aldo-keto reductase family 1 member B10 (AKR1B10) is indispensable to cancer development and progression, which has served as a diagnostic biomarker for tumors. In our study, we demonstrated that the expression of AKR1B10 in GC tissues was significantly lower compared with normal gastric tissues. Subgroup analysis showed that, according to the clinic-pathological factors, the effect of the AKR1B10 expression level on the prognosis of GC patients was significantly different. Moreover, reduced expression of AKR1B10 promoted the ability of GC cells in proliferation and migration. Furthermore, increased AKR1B10 levels resulted in the opposite trend *in vitro*. Moreover, AKR1B10 was correlated with epithelial-mesenchymal transition (EMT) in a significant way. *In vivo* experiment, knockdown of AKR1B10 promoted the growth of tumor, increased Vimentin, and E-cadherin significantly. In summary, AKR1B10 is considered as a tumor suppressor in GC and is a promising therapeutic target.

## INTRODUCTION

Gastric cancer (GC) is one of the most common digestive malignancies around the world [1], especially in East Asia such as China. Due to continuous development in the diagnosis and treatment of GC, the incidence of GC is gradually decreasing, and in particular, the number of cases of early gastric cancer among all the cases of GC is rapidly on the rise. What we urgently need is to explore the development of GC and identify novel therapeutic targets. Aldo-keto reductase family 1 member B10 is a 36-kD cytosolic NADPH-dependent oxidoreductase [2]. AKR1B10 is mainly expressed in digestive tract tissues such as the stomach, small intestine, and colorectum. In the liver, thymus and prostate, the expression can be low, but in other normal tissues the expression is zero [2–5]. AKR1B1 and AKR1B10 have a similar structure but the functions are different [6, 7]. AKR1B1 participates in the conversion of glucose into sorbitol. Thus, this enzyme has been found to be involved in the pathophysiological processes of diabetes. The effect of AKR1B10 on glucose is not known, but it has been proven to be involved in the metabolism of 4-hydroxynonenal, acrolein and phospholipid aldehydes [8, 9]. Increasingly researches have demonstrated that AKR1B10 is indispensable to development of GC, and targeted therapy for AKR1B10 may be an effective treatment regimen. As reported, AKR1B10 has been shown to be secreted via nonclassical pathway mediated by lysosome [10]. In addition, it is well known to be overexpressed in human pancreatic cancer, hepatocellular carcinoma, and lung cancer [2, 8, 11, 12]. However, most studies on the effects of AKR1B10 on tumors have focused on non-gastrointestinal tumors. The role of gastrointestinal tumors has not yet been elucidated.

The ability of GC cells in proliferation and metastasis are caused by abnormal activation of epithelial-mesenchymal transition (EMT), which is activated by factors such as TGF-β and EMT inducers, including Snail, while some inducers, such as α-SMA have opposite regulatory effects [13, 14]. Our findings demonstrated that EMT of GC cells was likely regulated by AKR1B10. Furthermore, alteration of AKR1B10 level affected characteristics of GC cells such as proliferation and migration.

Due to differential expression of AKR1B10 in gastrointestinal tumors and other tumors, the feasibility of targeted therapy for AKR1B10 is not clear [6, 8, 11, 15]. Few studies demonstrated the value of AKR1B10 in diagnosing, estimating prognosis as well as impacting the functional behavior of tumor. Additional studies on whether AKR1B10 can be a potential target for gastrointestinal cancer therapy are critical.

## MATERIALS AND METHODS

### Human tissue specimens

Human GC and adjacent normal tissues were collected immediately after radical surgical resection. Total 117 paired specimens were collected from 2010 to 2012. All samples were obtained with informed consent from patients who had not undergone radiotherapy or chemotherapy prior to surgery. All specimens were histo-pathologically verified. In accordance with the guidelines of Independent Ethics Committee of the Affiliated Suzhou Hospital of Nanjing Medical University (IRB approval number, KL901066) (Nanjing, China), the study was approved and all patients signed informed consent.

### Immunohistochemistry (IHC) evaluation

10% formalin was used to fix the tumor tissues, after embedding, the tissues were continuously cut into 5-μm-thick slices. Sections were dewaxed, rehydrated, quenched with hydrogen peroxide in methanol, and blocked with 10% normal goat serum for 30 min. After blocking, per sample was incubated overnight with a 1:100 dilution polyclonal anti-human AKR1B10 (ABclonal, China) at 4°C or at room temperature for 2 hours. In accordance with manufacturer's protocol, processed tissues were immunostained using tissue staining kit (Zhongshan Biotechnology, China) and scored by two authors respectively [16].

### Bioinformatics analysis

AKR1B10 expression and the effect it exerts on the prognosis were analyzed by the GEPIA (http://gepia.cancer-pku.cn) and Oncomine (http://www.oncomine.org) platform. The level of AKR1B10 in GC cell was searched from CCLE (https://portals.broadinstitute.org/ccle) platform. All the information obtained was analyzed by established protocols.

### Cell culture and transfection

Cell Bank of Chinese Academy of Sciences (Shanghai, China) was the source of GC cell lines (MKN45 and AGS). At 37°C under 5% CO_2_, MKN45 and AGS cells were cultured in RPMI 1640 medium (Hyclone, USA), supplemented with 10% fetal bovine serum (Gibco, USA). Specific ingredients include penicillin G sodium (100 U/ml) and streptomycin (100 μg/ml). The prepared human AKR1B10-shRNA was transfected into MKN45 cells. AGS cells were processed by AKR1B10 cDNA plasmid [17].

### Protein isolation and western blot analysis

As manufacturer’s protocol, GC cells were lysed in ice-cold RIPA lysis buffer supplemented with protease and phosphatase inhibitors. Extracted proteins were separated by SDS-PAGE and transferred onto PVDF membranes (Millipore, USA).

After blocking with 5% non-fat milk for 1 h, membranes were incubated overnight with antibodies at 4°C. Protein bands were visualized by chemiluminescence and quantified by ImageJ for Windows (NIH, USA). Specific antibodies include: anti-AKR1B10 (1:1000; no. bs-6274R; Bioss), anti-E-cadherin (1:1000; no. bs-1016R; Bioss), anti-vimentin (1:1000; no. bs-23063R; Bioss) and anti-GAPDH (1:5000; no. bs-0755R; Bioss).

### RNA isolation and quantitative real-time PCR (qRT-PCR)

Total RNA of GC tissues or cells was extracted by TRIzol reagent (Invitrogen, Life Technologies, USA). After reverse transcription by a RevertAid First Strand cDNA Synthesis Kit (Thermo Fisher Scientific, USA), as manufacturer’s instructions, qRT-PCR was conducted by Power SYBR^®^ Green PCR Master Mix (ABI, USA) and 7500 real time PCR system (ABI, USA).

β-actin was used as the internal control. The primer sequences were as follows: AKR1B10 forward (5′-CCCAAAGATGATAAAGGTAATGCCATCGGT-3′) and reverse (5′-CGATCTGGAAGTGGCTGAAATTGGAGA-3′); E-cadherin forward (5′-CGGGAATGCAGTTGAGGATC-3′) and reverse (5′-AGGATGGTGTAAGCGATGGC-3′); Vimentin forward (5′-GAGAACTTTGCCGTTGAAGC-3′) and reverse (5′-GCTTCCTGTAGGTGGCAATC-3′); β-actin forward (5′-CCACACTGTGCCCATCTACG-3′) and reverse (5′-AGGATCTTCATGAGGTAGTCAGTCAG-3′).

### CCK-8 assay

Cell suspension was inoculated in 96-well plates, and CCK8 solution (APExBIO, USA) was added to each well. After incubation, absorbance was measured at 450nm with a microplate reader, and then cell proliferation inhibition rate was calculated. All samples were performed in triplicate.

### Colony formation assay

After transfection, 1000 experimental cells were seeded in each well of 6-well plates. After 10 days cultivation, the cells were fixed and stained with 0.1% crystal violet. By means of optical microscope (Nikon, Japan) that was equipped with digital camera (Nikon, Japan), we counted the number of colonies at 40x magnification.

### Cell migration assay

Detection of cell migration via Transwell plates (Corning Incorporated, USA) and by means of a microscope at 200× magnification to observe it [17]. Five fields were selected per specimens for quality statistics. The final result was calculated after removing one maximum and minimum value.

### Subcutaneous xenograft establishment

Male BALB/c nude mice (3-5-week old, 16-18 g, specific pathogen-free grade) were obtained from Shanghai SLRC Laboratory Animal Co. Ltd. (Shanghai, China). Mice were randomly divided into AKR1B10 knock down (KD) and negative control (NC) groups (*n* = 5). 5 × 10^6^ AKR1B10-KD or NC-shRNA MKN45 were injected into left and right dorsal flank on day 0 subcutaneously [18]. Animal experiments were approved by the Animal Ethics Committee of the Affiliated Suzhou Hospital of Nanjing Medical University (Nanjing, China).

### Statistical analysis

Different data were compared using chi-squared, Student's *t*-test (unpaired, two-tailed), Mann–Whitney *U* test or one-way ANOVA. All data were presented as the mean ± SD. Moreover, Kaplan-Meier curves were plotted for survival analysis. SPSS version 25.0 (IBM, USA), R programs (https://cran.r-project.org) and GraphPad Prism 8 (USA) were conducted to analyze statistics. *P* < 0.05 was considered statistically significant.

## RESULTS

### AKR1B10 is upregulated in GC tissues and associated with clinico-pathological factors

AKR1B10 expression levels were searched in multiple cancer types via the GEPIA Platform. Compared with para-tumor tissues, the levels of AKR1B10 in GC tissues were decreased in a significantly way (Supplementary Figure 1A–1B). Furthermore, in a certain of non-gastrointestinal tissues, the expression of AKR1B10 in most tumor tissues was higher in comparison to normal tissues (Supplementary Figure 1A). High levels of AKR1B10 may predict prolonged disease-free survival (DFS) in the TCGA dataset by GEPIA significantly (Supplementary Figure 1C), while an overall survival (OS) was not affected by AKR1B10 expression (Supplementary Figure 1D). The results of the Oncomine platform showed that, compared to non-tumor tissues, AKR1B10 mRNA levels in GC tissues were increased in Cui and DErrico datasets (Supplementary Figure 1E). For further analysis, 5 datasets of Oncomine platform were used to compare AKR1B10 mRNA expression in GC and normal tissues. The results revealed a similar expression trend (Supplementary Figure 1F).

**Figure 1:**
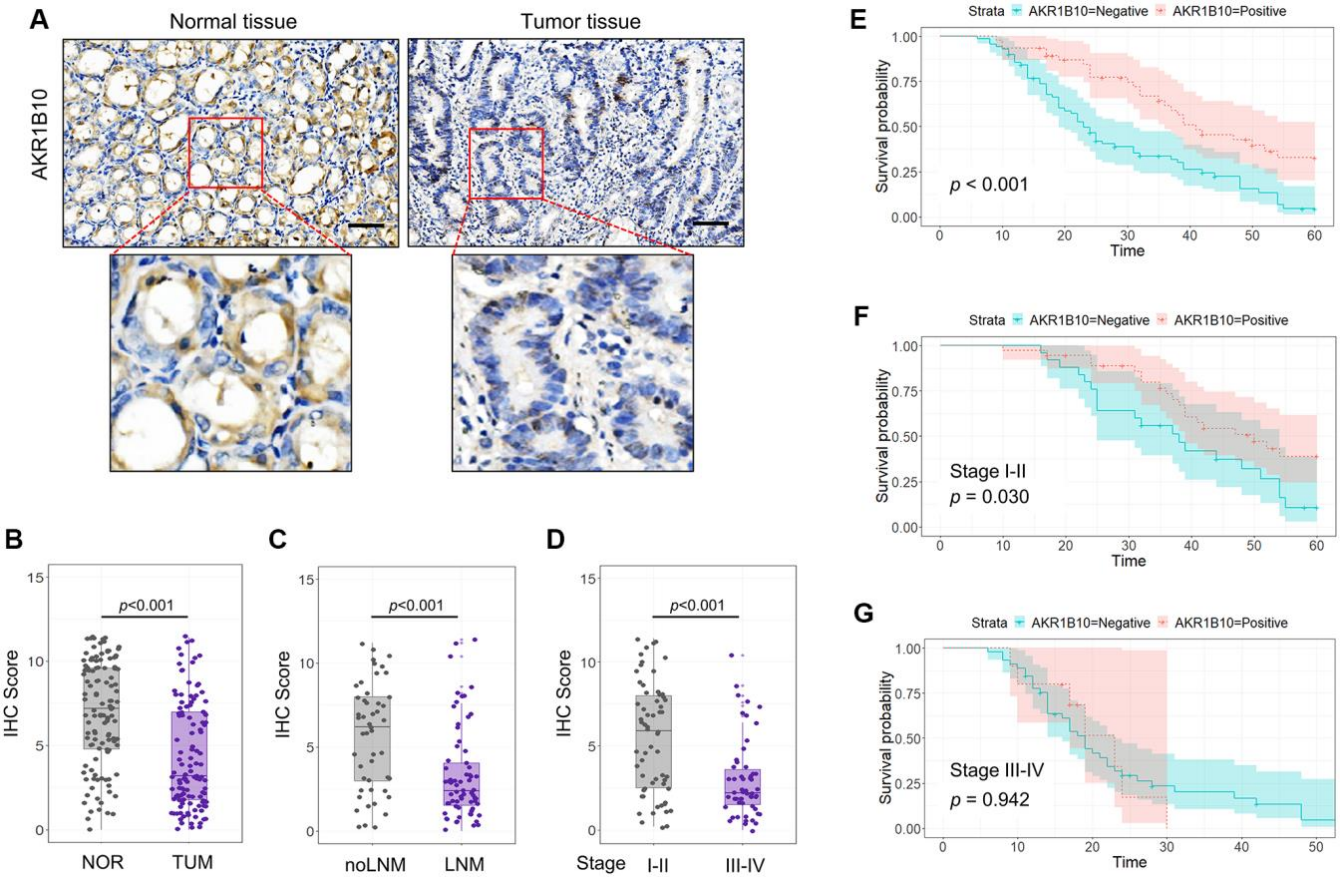
**Expression of AKR1B10 in gastric cancer tissues.** (**A**) Representative immunohistochemistry images showing *in situ* AKR1B10 expression in gastric cancer (GC) and normal tissues (scale bar = 100 μm). (**B**–**D**) IHC scores of AKR1B10 in (**B**) GC vs normal tissues, (**C**) tumors with and without lymph node invasion, and (**D**) TNM stage I–II vs III–IV. (**E**–**G**) overall survival analysis of (**E**) AKR1B10^pos^ vs AKRiB10^neg^ GC patients, and in subgroups overall survival analysis of TNM stage I–II (**F**) and III–IV (**G**). LNM, Lymph node metastasis.

To confirm the expression of AKR1B10 in GC, 117 specimens were analyzed for AKR1B10 expression (Figure 1A). Quantification of IHC scores indicated a significant reduction about AKR1B10 levels in GC compared with normal gastric tissues (Figure 1B, Supplementary Figure 2). In addition, AKR1B10 decreased in patients with lymph node metastasis in a significant way compared with patients without (Figure 1C). Furthermore, the expression of AKR1B10 was related to stage of tumor, that is, the expression level in tumor-node-metastasis (TNM) stage III–IV was lower than that in I–II (Figure 1D). Datasets from the GEPIA and Oncomine platforms further validate our results.

A summary about AKR1B10 and clinico-pathological characteristics was made in Table 1. The expression of AKR1B10 had significant association with tumor size (*P* < 0.001), depth of invasion (*P* < 0.001), lymph node metastasis (*P* < 0.001), venous invasion (*P* = 0.002), and TNM stage (*P* < 0.001), while no correlation with other clinicopathological variables such as age, gender, degree of differentiation or neural invasion (*P* > 0.05). Additionally, AKR1B10 can be used as a single factor to predict univariate risk, determined by Cox's proportional hazard model analysis (Table 2).

**Table 1:** Relationship between AKR1B10 and clinic-pathological factors in GC patients.

**Variables**	**AKR1B10**		
	**Negative**	**Positive**	***P* value**
Age (years)			
≤60	25	19	0.606
>60	45	28	
Gender			
Male	53	37	0.705
Female	17	10	
Tumor size (cm)			
<5	38	41	<0.001^*^
≥5	32	6	
Depth of tumor invasion			
T1–2	10	21	<0.001^*^
T3–4	60	26	
Lymph node metastasis			
No	18	31	<0.001^*^
Yes	52	16	
Degree of differentiation			
Well	32	27	0.213
Poor	38	20	
Venous invasion			
Negative	39	39	0.002^*^
Positive	31	8	
Neural invasion			
Negative	43	35	0.142
Positive	27	12	
TNM staging			
I–II	25	37	<0.001^*^
III–IV	45	10	

^*^P < 0.05.

**Table 2:** Results of univariate and multivariate analyses of postoperative patients’ survival by Cox’s proportional hazard model.

**Varieties**	***n***	**Univariate analysis**			**Multivariate analysis**		
		**HR**	**95% CI**	***p* value**	**HR**	**95% CI**	***p* value**
Age (≤60 or > 60 years)	44/73	0.796	0.503–1.259	0.330			
Gender (Male/Female)	90/27	0.902	0.540–1.507	0.694			
Size of tumor (≤5 or > 5 cm)	79/38	0.356	0.226–0.560	<0.001^*^	0.795	0.464–1.361	0.402
Depth of tumor invasion (T1–2/T3–4)	31/86	0.241	0.137–0.421	<0.001^*^	0.429	0.211–0.873	0.020
Lymph node metastasis (negative/positive)	49/68	0.271	0.168–0.437	<0.001^*^	0.498	0.233–1.064	0.072
Degree of differentiation (moderate-well/poor)	59/58	0.576	0.374–0.887	0.012^*^	0.81	0.503–1.304	0.385
Venous invasion (negative/positive)	78/39	0.384	0.247–0.597	<0.001^*^	0.725	0.429–1.226	0.231
Neural invasion (negative/positive)	78/39	0.466	0.297–0.729	0.001^*^	1.182	0.691–2.023	0.541
TNM staging (I–II/III–IV)	62/55	0.243	0.153–0.385	<0.001^*^	0.832	0.394–1.759	0.631
AKR1B10 expression (negative/positive)	70/47	2.401	1.504–3.832	<0.001^*^	1.161	0.673–2.004	0.591

^*^P < 0.05.

### Maintained level of AKR1B10 improved prognosis of GC patients

In accordance with IHC scores, patients who expressed AKR1B10 positive or negative were divided into different subgroups. Results indicated the fact that AKR1B10^neg^ patients suffered worse post-surgery overall survival than that of AKR1B10^pos^ patients (Figure 1E). Interestingly, In patients with TNM stages I–II, maintained AKR1B10 expression was associated with favorable in patients with TNM stages I–II but not stage III–IV patients (*P* = 0.030; *P* = 0.942; Figure 1F–1G).

**Figure 2:**
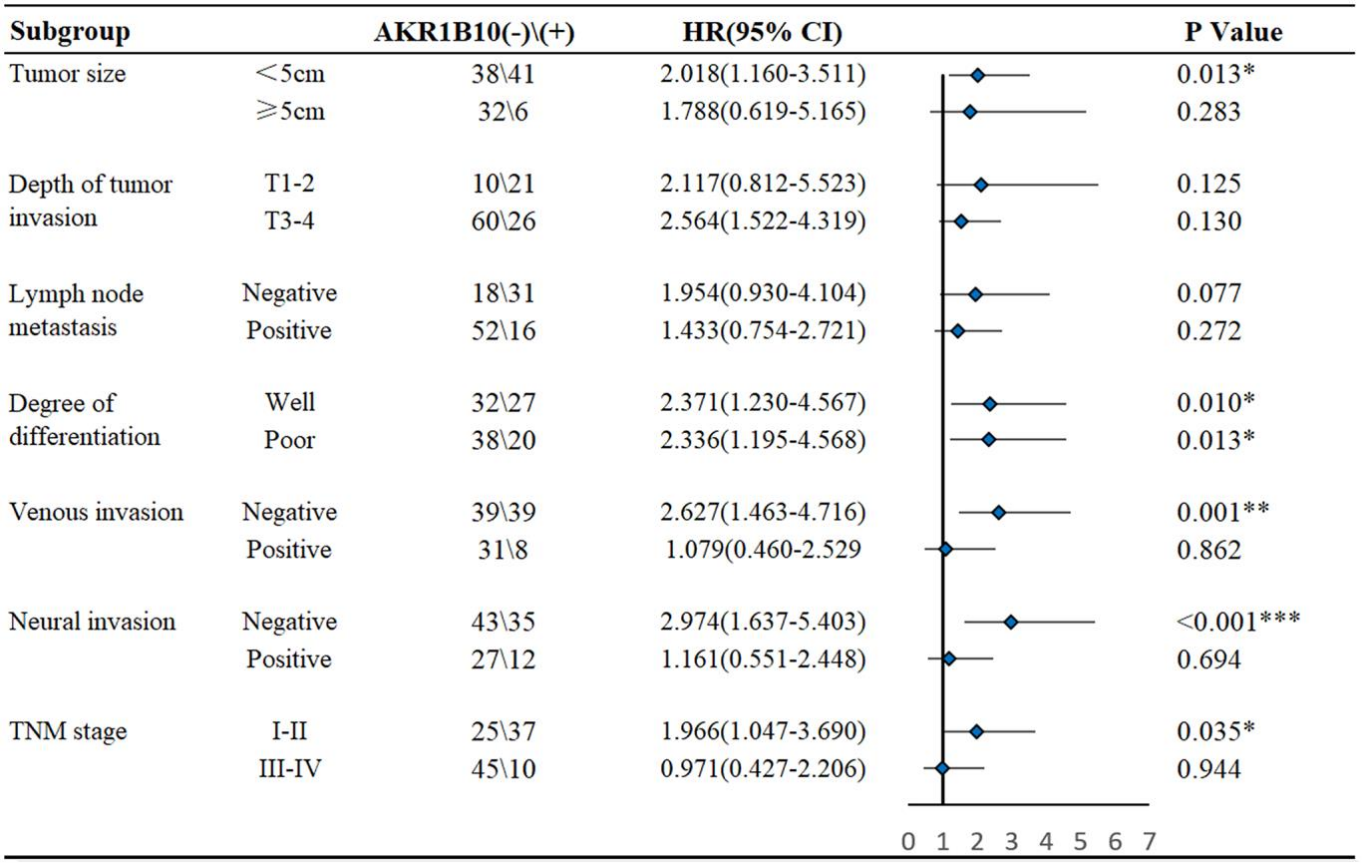
Subgroup analysis of the influence of AKR1B10 expression on the survival of gastric cancer patients.

For further elucidate the value of AKR1B10 in prognosis, patients were divided into different subgroups according to AKR1B10 expression levels (Figure 2). No matter how degree of infiltration depth, lymph node metastasis and differentiation vary, AKR1B10 expression exerted no obvious effect on the prognosis of GC patients. However, AKR1B10^neg^ patients with smaller tumor size (*P* = 0.013), negative venous invasion (*P* = 0.001), negative neural invasion (*P* < 0.001) and TNM stage I–II (*P* = 0.035) faced a worse prognosis. Conversely, for patients with tumor size larger than 5cm (*P* = 0.283), venous invasion (*P* = 0.862), neural invasion (*P* = 0.694), and TNM stage III–IV (*P* = 0.944), the impact of AKR1B10 on disease prognosis is insignificant.

**Figure 3:**
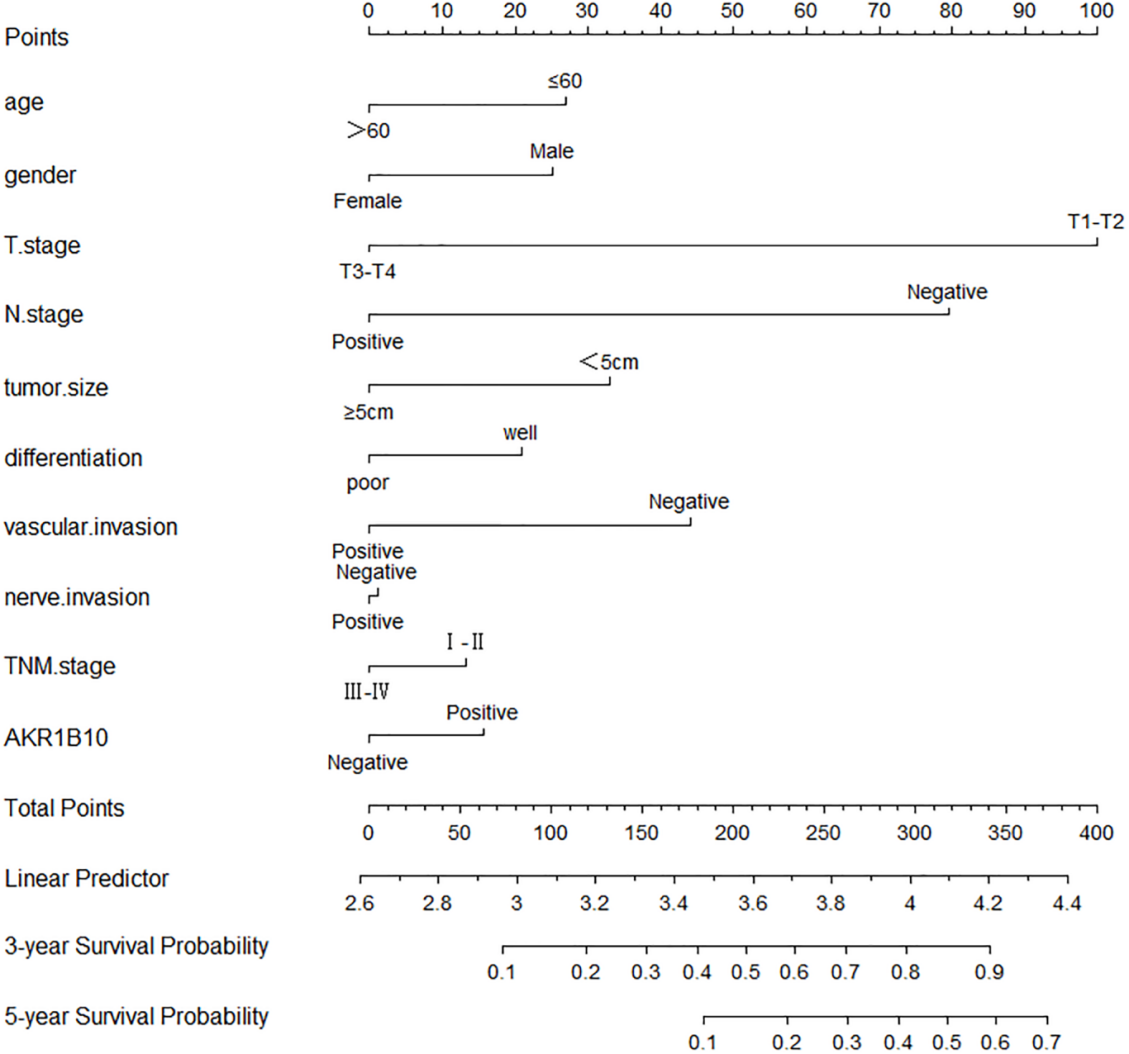
**Nomograms to predict survival of gastric cancer patients.** Points of each variable were obtained via a vertical line between each variable and the point scale. The predicted survival rate was correlated with the total points by drawing a vertical line from the total points scale to the overall survival.

Age, gender, tumor size, differentiation, vascular invasion, neural invasion, TNM stage, and AKR1B10 expression were applied to evaluate 3- and 5- year OS. The nomogram gave every prognostic variable a score on the point scale. We obtained scores associated with each prognostic variable and calculated the overall score (Figure 3).

### Increased AKR1B10 expression inhibits the ability of GC cells in proliferation and migration

**Figure 4:**
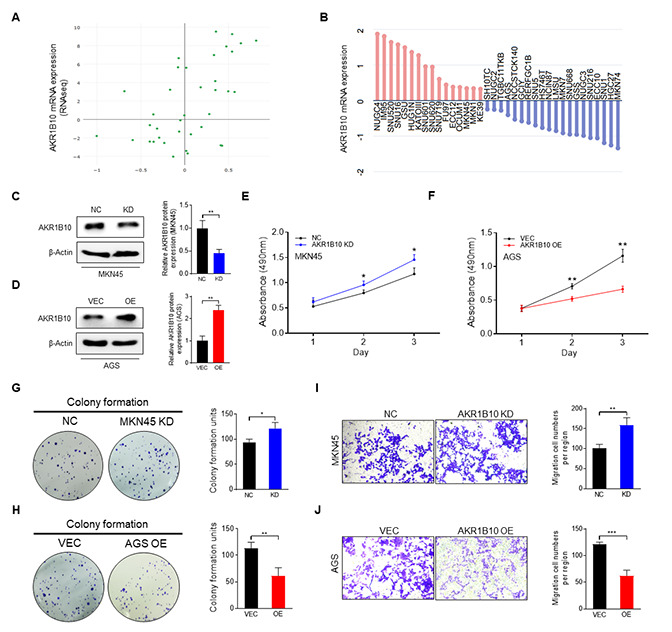
**Effect of AKR1B10 on the cell proliferation and migration ability of gastric cancer cells.** (**A**) AKR1B10 expression in gastric cancer (GC) cell lines from the CCLE platform. (**B**) Relative expression of AKR1B10 in GC cell lines according to RNAseq results via the CCLE platform. (**C**–**D**) Immunoblot showing AKR1B10 protein levels in MKN45 cells transfected with AKR1B10-shRNA (**C**) and in AGS cells transfected with the AKR1B10 overexpression plasmid (**D**), and gray value analysis via ImageJ. (**E**–**J**) Proliferation rates (**E**–**F**), colony forming ability (**G**–**H**), and migration ability (**I**–**J**) of AKR1B10-KD and AKR1B10-OE GC cells. CCLE, Cancer Cell Line Encyclopedia. NC, negative control. KD, knockdown, AKR1B10-shRNA. VEC, vector. OE, overexpression, AKR1B10 overexpression plasmid. Data are presented as the mean ± SD (*n* = 3). ^*^P < 0.05, ^**^P < 0.01, ^***^P < 0.001.

To further elucidate the link between AKR1B10 and GC cells, we determined the AKR1B10 expression level of GC cells by the CCLE platform (Figure 4A–4B).

Due to the fact that AKR1B10 expressed highly in MKN45 cells and low in AGS, MKN45 cells were transfected with control- or AKR1B10-shRNA to knockdown AKR1B10. AGS cells were transfected with vector or AKR1B10 overexpression plasmid to amplify AKR1B10 (Figure 4C–4D). For further analyzed the biological characteristics of AKR1B10, we conducted knockdown (KD) and overexpression (OE) constructs. What we found is that AKR1B10-KD increased cellular proliferation ability, whereas that of AKR1B10-OE was reduced (Figure 4E–4F). Consistent with these findings, AKR1B10-KD cells strengthened colony-formation (Figure 4G–4H) and migration (Figure 4I–4J) ability, whereas AKR1B10-OE cells indicated reversed impacts.

### Expression of AKR1B10 is associated with the EMT in GC

**Figure 5:**
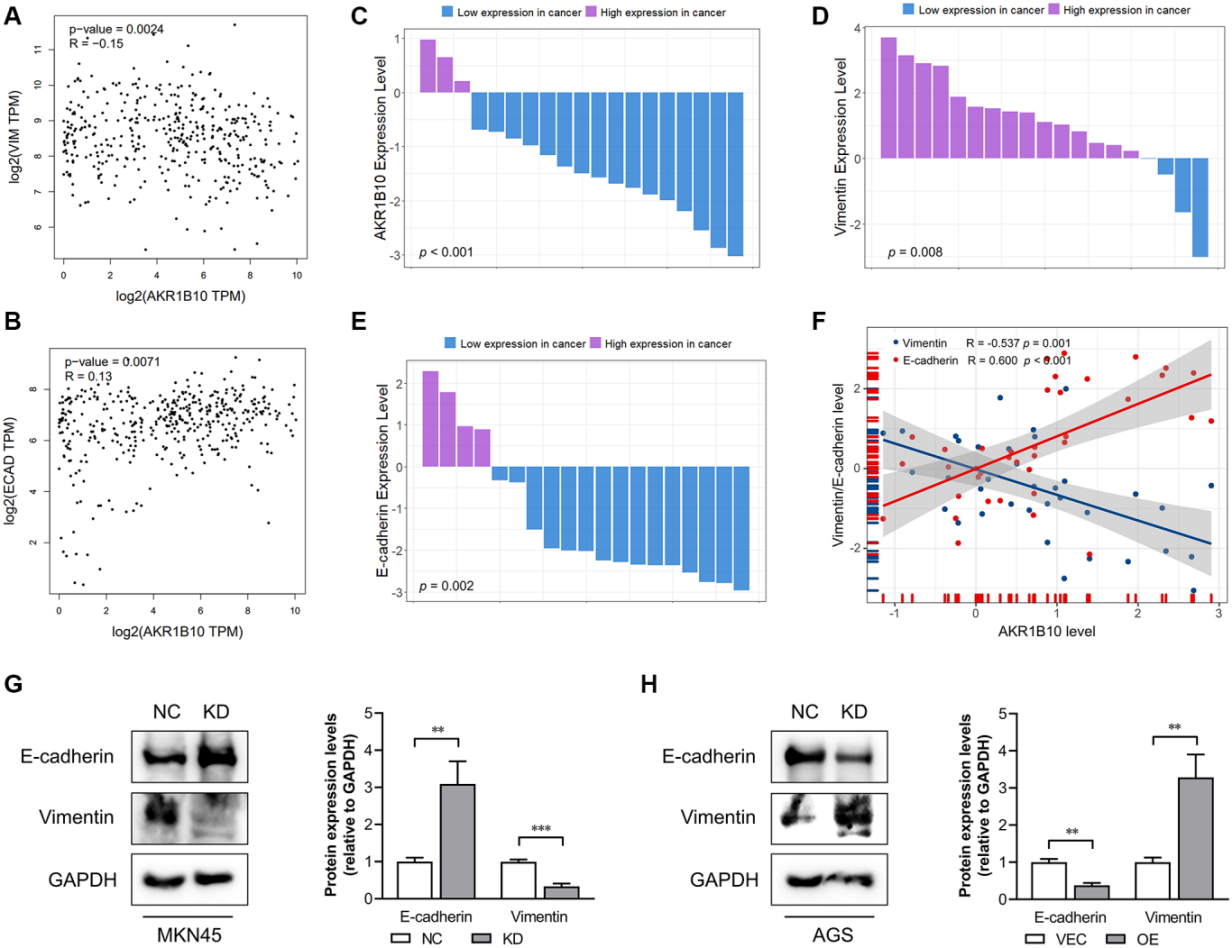
**Correlation between AKR1B10 and epithelial-mesenchymal transition.** (**A**) Correlation analysis of AKR1B10 and Vimentin gene expression levels in gastric cancer (GC) patients by the GEPIA platform. (**B**) Correlation analysis of AKR1B10 and E-cadherin gene expression levels in GC patients by the GEPIA platform. (**C**–**E**) Comparison of AKR1B10 (**C**), Vimentin (**D**) and E-cadherin (**E**) mRNA levels in 19 paired GC and normal tissues. (**F**) Correlation between AKR1B10 and Vimentin, and between AKR1B10 and E-cadherin mRNA levels in GC tissues. (**G**) MKN45 cells transfected with NC or KD and (**H**) AGS transfected with VEC or AKR1B10-OE. The bands were semi-quantified by ImageJ and the results are presented as the mean ± SD. VIM, Vimentin; ECAD, E-cadherin; TPM, transcripts per million. NC, negative control; KD, knockdown; VEC, vector; OE, overexpression. ^**^P < 0.01, ^***^P < 0.001.

AKR1B10 regulated the ability of GC cells to proliferate and migrate negatively. However, EMT is closely related to migration of tumor cells. Therefore, what deserves further determination is whether AKR1B10 affect EMT of tumor or not. Correlation between AKR1B10 mRNA expression and EMT-related gene expression was analyzed in GC specimens from the TCGA dataset by the GEPIA platform. Specifically speaking, between AKR1B10 mRNA expression and EMT-related genes, including Vimentin (Figure 5A), Snail family transcriptional repressor 1, 2 (SNAI1, 2 Supplementary Figure 3A, 3B), zinc finger E-box binding homeobox 1, 2 (ZEB1, 2 Supplementary Figure 3C, 3D), signified a negative correlation, whereas correlated with E-cadherin positively (Figure 5B).

Next, we assessed AKR1B10, Vimentin, and E-cadherin mRNA levels in GC tissues. Compared with 19 paired normal gastric tissues, AKR1B10 expression was downregulated in GC tissues (Figure 5C, Supplementary Figure 4A–4B). Moreover, Vimentin was elevated and E-cadherin was reduced in GC tissues (Figure 5D–5E, Supplementary Figure 4C–4F). According to the results of AKR1B10, Vimentin and E-cadherin mRNA level in 19 GC tissues, AKR1B10 indicated a negative correlation with Vimentin while correlated with E-cadherin positively (Figure 5F). Additionally, the detection of E-cadherin and Vimentin were conducted by immunoblot analysis (Figure 5G). AKR1B10 silencing significantly reduced the expression of E-cadherin and increased that of Vimentin. In contrast, AKR1B10 overexpression affected expression of EMT markers oppositely (Figure 5H). Taken together, it is not difficult to draw conclusion that AKR1B10 is crucial to EMT in GC cells.

### AKR1B10 inhibits gastric tumorigenesis *in vivo*

**Figure 6:**
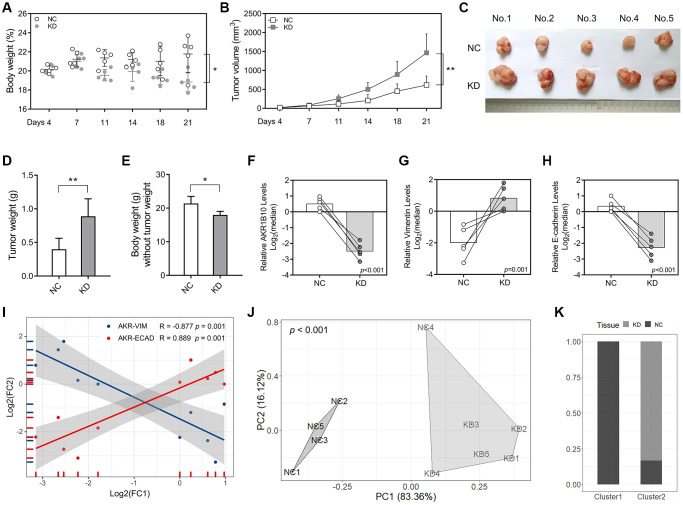
**AKR1B10 knockdown promotes gastric cancer tumor growth *in vivo*.** (**A**–**B**) Total body weight (**A**) and tumor volume (**B**) of the mice. (**C**) Representative pictures of subcutaneous tumors harvested from NC and AKR1B10-KD group. (**D**) The weights of tumor masses. (**E**) The weights of mice without tumor masses. (**F**–**I**) Relative AKR1B10 (**F**), Vimentin (**G**) and E-cadherin (**H**) mRNA levels in tumors of the AKR1B10-KD or NC group, and their correlation (**I**). (**J**) Stratification of mice into cluster 1 and cluster 2 according to AKR1B10 mRNA levels, body weight and tumor weight. (**K**) Percentage of NC and AKR1B10-KD mice in each cluster. Data are presented as the mean ± SD. NC, negative control; KD, AKR1B10 knockdown. ^*^P < 0.05, ^**^P < 0.01.

For further analyze how AKR1B10 impacted GC cell growth, wild-type and AKR1B10-KD MKN45 cells were used as an *in vivo* xenograft model. Depletion of AKR1B10 exerted the reduction of body weight in mice (Figure 6A), and facilitated GC cells proliferation, which was manifested by the fact that tumor size (Figure 6B–6C) and weight (Figure 6D) were greater than that of NC group. The difference in body weight between AKR1B10-KD and control group without tumors was obvious (Figure 6E). Additionally, *in situ* mRNA levels of AKR1B10 and E-cadherin indicated a reduction in a significant way, whereas Vimentin was higher in AKR1B10-KD group (Figure 6F–6H). Results showed a significant correlation (Figure 6I).

Furthermore, we conducted cluster analysis in order to explore whether AKR1B10 levels, body weight and tumor weigh had combined effect. Mice in the AKR1B10-KD group and the control group were significantly differentiated (Figure 6J–6K).

## DISCUSSION

The AKR1B subgroup is associated with numerous diseases, such as diabetes and cancers. AKR1B10 is one of the subgroups included in AKR1B [2]. The expression of AKR1B10 is primarily distributed in digestive tract, meaning that the expression in non-digestive tract tissues is low. Moreover, what calls for special attention is that the level of AKR1B10 is opposite in tumors. It was found that AKR1B10 has enzymatic activity for substrates including retinaldehyde [19] and lipid peroxidation products [20].

AKR1B10 is indispensable to the development of tumors, becoming a diagnostic biomarker for some tumors. Inhibiting AKR1B10 may be an ideal treatment strategy [21, 22]. An AKR1B10 inhibitor used widely in tumor treatment has recently attracted increased attention [23–25]. AKR1B10 had statistical difference in gastrointestinal and non-gastrointestinal tissues. However, the differences were reversed in tumor tissues. These findings attracted the attention of researchers and resulted in a differential effect of the targeted inhibition of AKR1B10 on tumor cells [9, 26, 27].

The level of AKR1B10 in GC tissues was significantly lower compared with that in normal tissues, demonstrated by TCGA database analysis via the GEPIA platform and tissue detection. At the same time, we demonstrated that the expression of AKR1B10 in TNM stage III–IV patients or patients with lymph node metastasis was lower than that in control group in a significant way. In the subgroup analysis, the predicting value of AKR1B10 in prognosis of GC was significantly different. This indicated that target therapy may be dependent on the subgroup conditions, which may appropriately reduce the effective population and improve the therapeutic effect [18].

For a novel target that is conductive to prevent and treat cancer, increasingly attention has been paid on inhibitors of AKR1B10 [25, 28, 29]. The analysis of these inhibitors improves the understanding of the important features to be considered in the design of these compounds: the inhibitors must properly match the binding sites of AKR1B10 [8]. In the treatment of non-gastrointestinal tumors, targeted therapy of AKR1B10 has an excellent effect. AKR1B10 may promote cell proliferation and inhibit cell apoptosis by regulating oncogene expression. By contrast, the downregulation of AKR1B10 in tumor cells suppresses the growth and progression of tumor [4, 11]. Studies have shown that the down-regulation of AKR1B10 promotes apoptosis, mediated by mitochondrial dysfunction and oxidative stress [30–32]. In addition, targeting against AKR1B10 and inducing autophagy of tumor cells may effectively inhibit the growth of tumor cells. AKR1B10P1 is the pseudogene of oncogene AKR1B10 in hepatocellular carcinoma, noticed as being anomalistic transcribed preliminarily. AKR1B10P1 stably impacts SOX4, EMT by sponging miR-138 directly, which modulates the regulating gene of SOX4 post-transcriptionally. Moreover, the phosphorylation of p70S6K at T389 was found a consistent reduction, which is known as a direct target of mTOR. In addition to phosphorylation of several proteins involved in protein synthesis, p70S6K can also affect the phosphorylation of proteins about cell growth, proliferation and motility. It also participates in the process of EMT. Whether AKR1B10 plays a specific function in GC associated with the EMT process is poorly understood [33, 34]. In this paper, we explored whether inhibition of AKR1B10 could affect proliferation and migration of GC cells, which may depend on the EMT process.

However, target treatment of AKR1B10 in gastrointestinal tumors has not been thoroughly studied, and the effect of AKR1B10 in gastrointestinal tumors has not been fully elucidated. Our study showed that in GC, inhibition of the expression level of AKR1B10 modestly promoted the capacity of tumor to proliferate, migrate and form colony. Furthermore, increased AKR1B10 expression can significantly inhibit the ability of GC cells in proliferation, migration, and colony formation. Therefore, we have the reason to believe that AKR1B10 impacts the proliferative and migratory ability of cells in gastrointestinal and non-gastrointestinal tumors reversely. In summary, the role of AKR1B10 in GC have not yet been fully elucidated. In addition, a accurate validation about the impact of AKR1B10 on growth of GC cells and tumor metastasis is still lacking. For the structure and function of AKR1B10, as well as AKR1B10 inhibitor interactions in GC deserve a comprehensive understanding, which may promote novel therapeutic strategies for GC especially combined with further studies.

## Supplementary Materials

Supplementary Figures
