# Supplementary material for: AKR1B10 inhibits the proliferation and migration of gastric cancer via regulating epithelial-mesenchymal transition
Source: Aging (Albany NY). 2021 Sep 22;13(18):22298–314. doi: 10.18632/aging.203538 (PMC8507292; doi:10.18632/aging.203538)
Supplement: Supplementary Figures [file aging-13-203538-s001.pdf]

SUPPLEMENTARY FIGURES

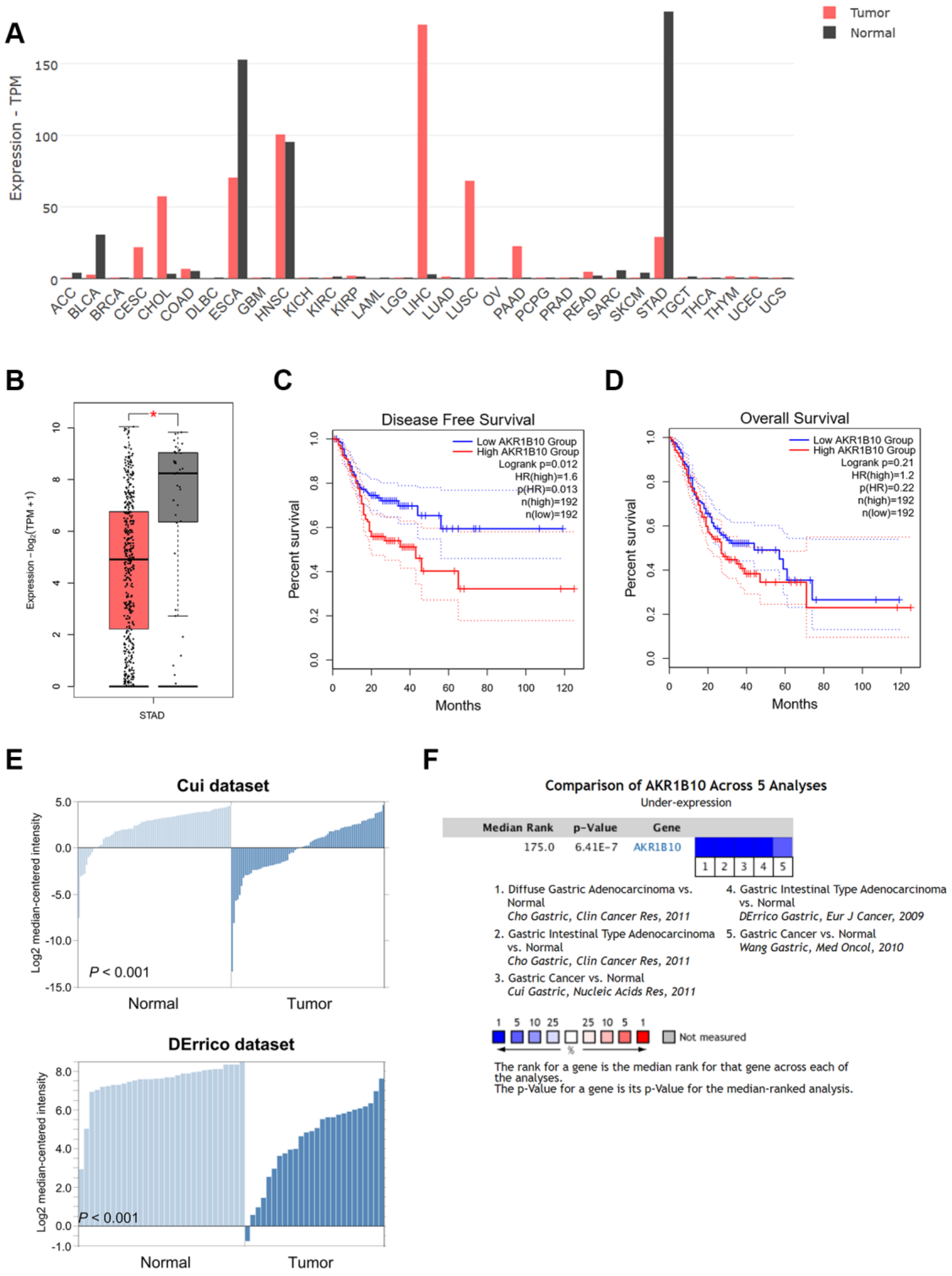

Supplementary Figure 1. Expression of AKR1B10 in gastric cancer in datasets. (**A**–**B**) Comparison of AKR1B10 levels in (**A**) multiple cancers, and (**B**) between gastric cancer (GC) and paired normal tissues in TCGA datasets via GEPIA platform. (**C**–**D**) disease free survival (**C**) and overall survival (**D**) were analyzed according to AKR1B10 expression level in GC tissues in the TCGA datasets via the GEPIA platform. (**E**) AKR1B10 mRNA levels in GC and non-tumor tissues in the datasets presented by the Oncomine platform. (**F**) Comparison of AKR1B10 mRNA expression in GC and normal tissues across 5 datasets by Oncomine platform.

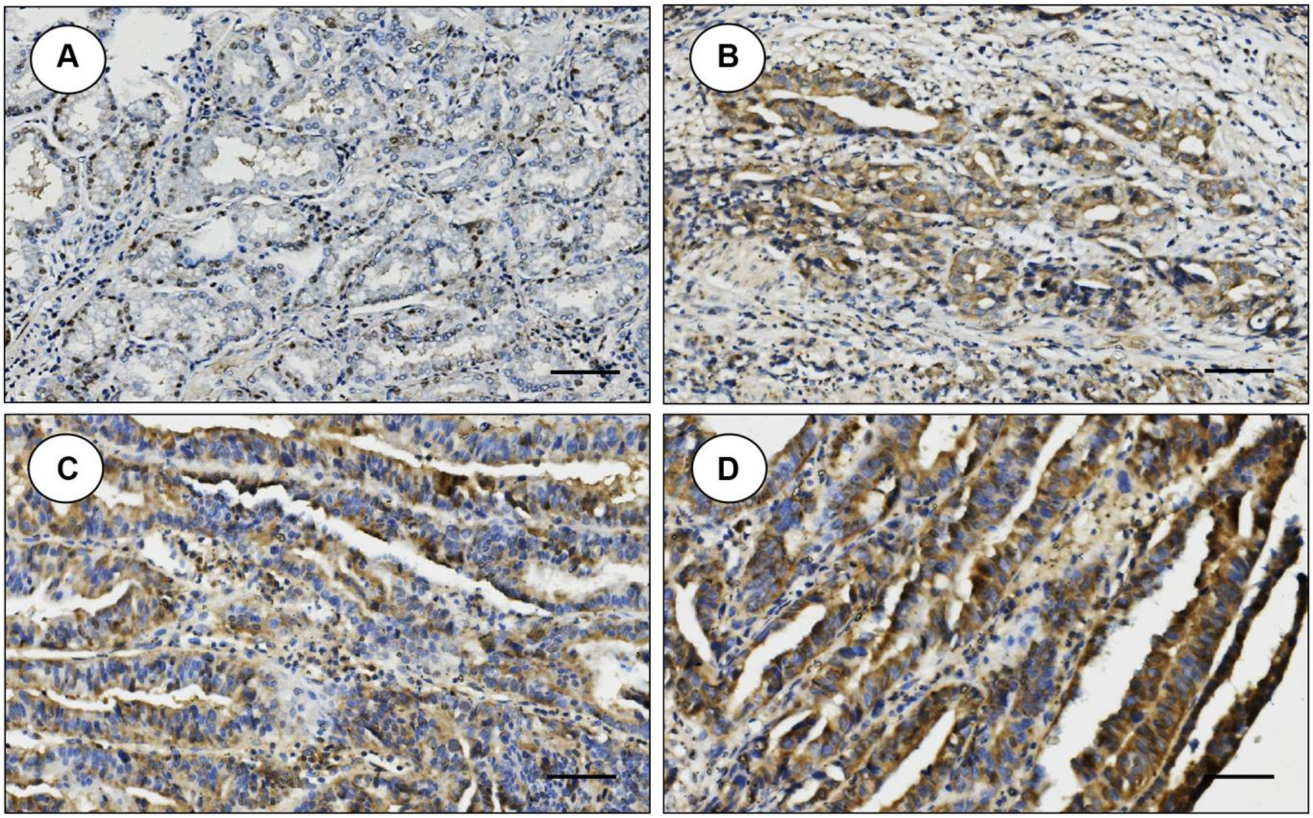

Supplementary Figure 2. Immunohistochemistry images showing *in situ* AKR1B10 expression in gastric cancer tissues. Negative (**A**), weak (**B**), positive (**C**), strong positive (**D**). Scale bar = 100 µm.

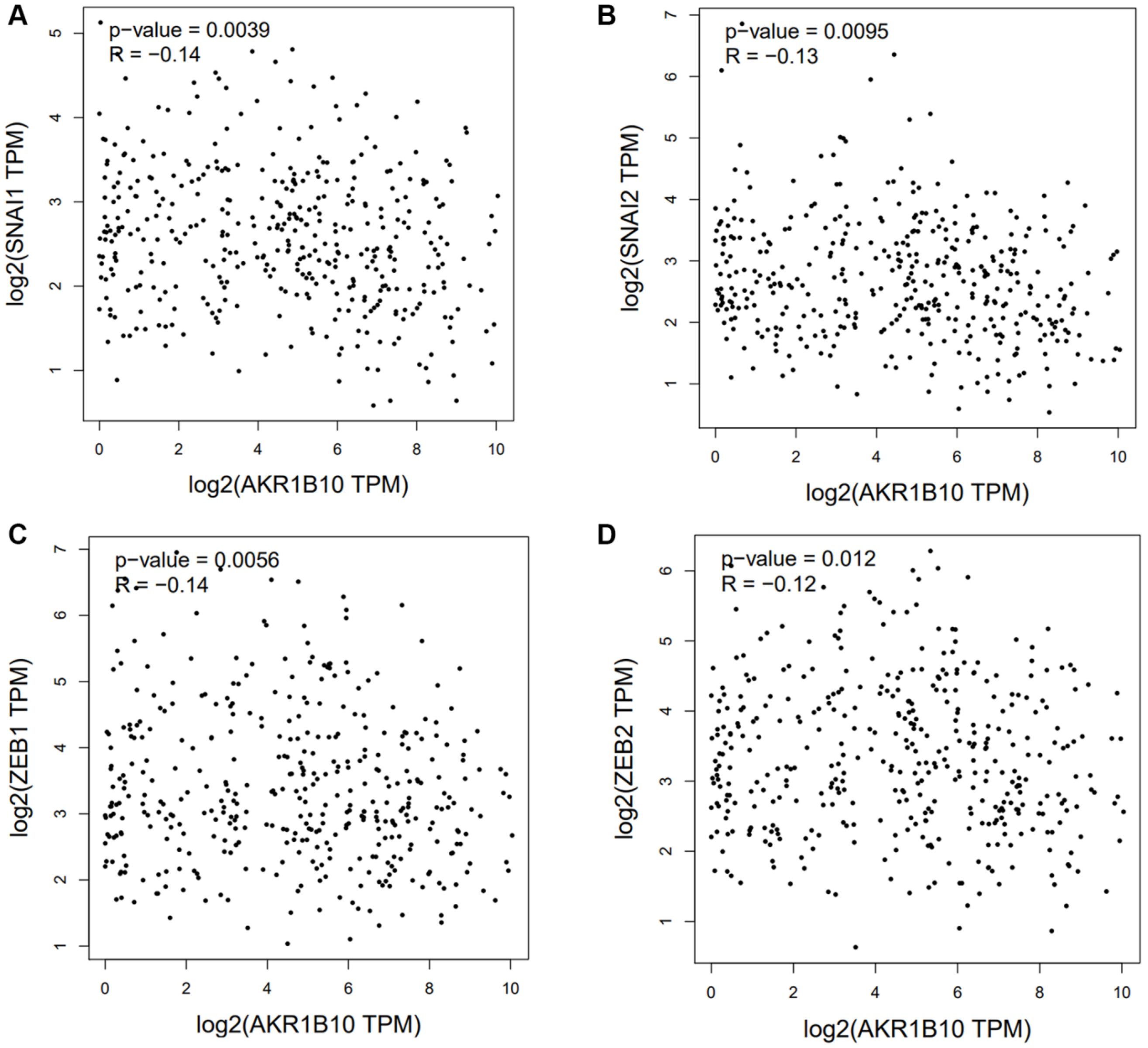

Supplementary Figure 3. AKR1B10 is associated with epithelial-mesenchymal transition. (**A**) Correlation analysis between AKR1B10 and SNAI1 gene expression in patients with gastric cancer (GC) by GEPIA datasets. (**B**) Correlation analysis between AKR1B10 and SNAI2 gene expression in patients with GC by GEPIA datasets. (**C**) Correlation analysis between AKR1B10 and ZEB1 gene expression in patients with GC by GEPIA datasets. (**D**) Correlation analysis between AKR1B10 and ZEB2 gene expression in patients with GC by GEPIA datasets. SNAI1/2, Snail family transcriptional repressor 1/2; ZEB1/2, zinc finger E-box binding homeobox 1/2; TPM, transcripts per million.

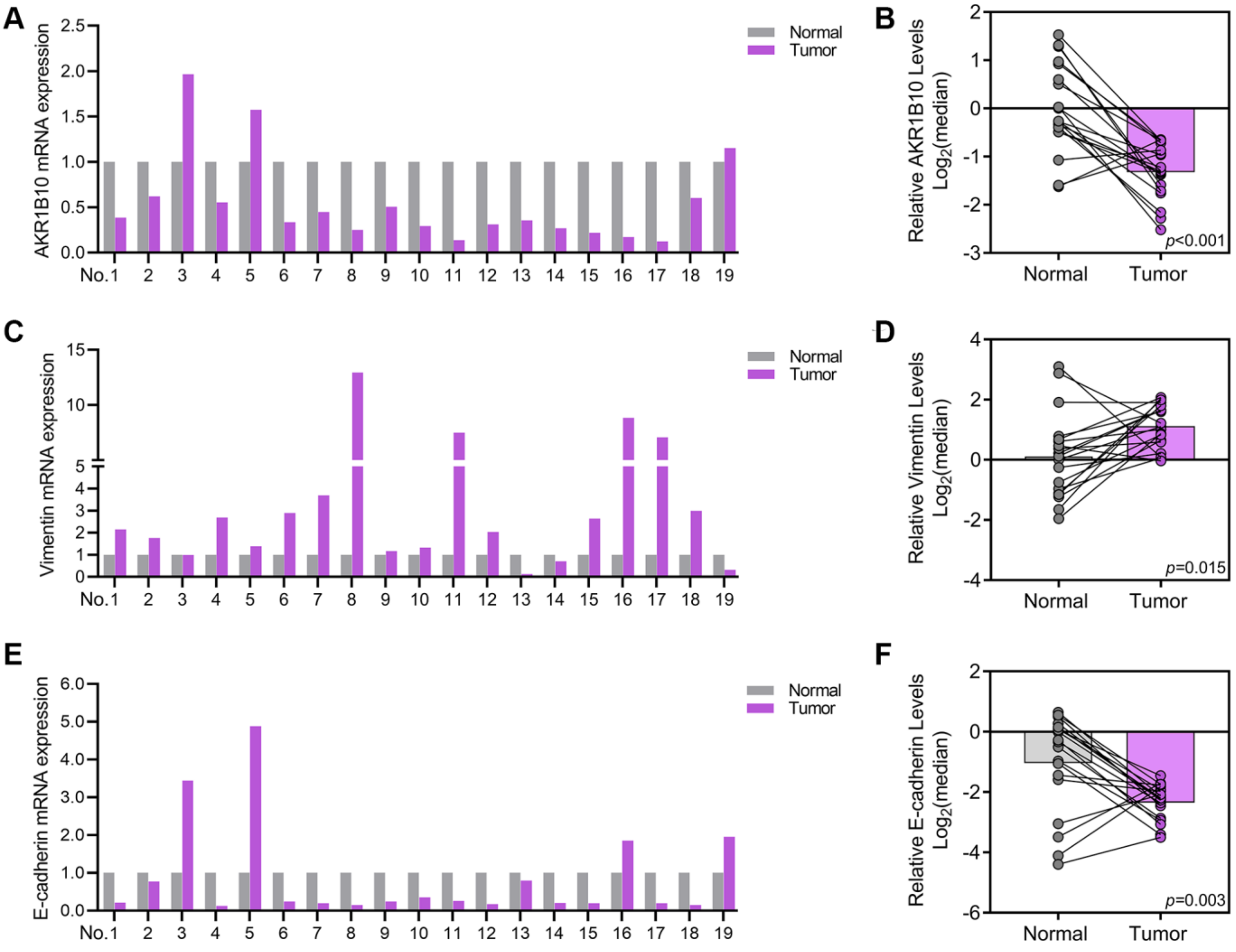

Supplementary Figure 4. mRNA level of AKR1B10, E-cadherin and Vimentin expression in gastric cancer (GC) and paired normal tissues. (**A**–**B**) AKR1B10 mRNA levels in 19 paired GC and normal tissues (**A**), comparison of AKR1B10 expression in paired GC and normal tissues (**B**). (**C**–**D**) Vimentin mRNA levels in 19 paired GC and normal tissues (**C**), comparison of Vimentin expression in paired GC and normal tissues (**D**). (**E**–**F**) E-cadherin mRNA levels in 19 paired GC and normal tissues (**E**), and comparison of E-cadherin expression in paired GC and normal tissues (**F**).
